# Experimental Study on Bending Fatigue Performance of ADI Gears Under Different Applied Load Levels

**DOI:** 10.3390/ma18163922

**Published:** 2025-08-21

**Authors:** Lijun Wang, Hui Wei, Hsinshen Ho, Bo Hu, Yangyang Li, Dongfei Wang

**Affiliations:** 1 School of Mechanical Engineering, North China University of Water Resources and Electric Power, Zhengzhou 450045, China; wanglijun@ncwu.edu.cn (L.W.); wh322419@163.com (H.W.); liyangyang7280@163.com (Y.L.); 2 School of Mechanical and Power Engineering, Zhengzhou University, Science Avenue 100, Zhengzhou 450001, China; hsinshen.ho@zzu.edu.cn (H.H.); hb17629355242@163.com (B.H.); 3 National Gear Product Quality Inspection and Testing Center, China Academy of Machinery Zhengzhou Research Institute of Mechanical Engineering Co., Ltd., Zhengzhou 450001, China

**Keywords:** ADI gears, bending fatigue testing, fatigue performance curves, metallographic analysis, crack analysis

## Abstract

As austempered ductile iron (ADI) is a key gear material for meeting the lightweight and cost-effective demands of new energy vehicles, its bending fatigue performance has a direct impact on vehicle transmission efficiency. In the present work, QTD 800 gears were subjected to bending fatigue testing using a combination of the conventional group method and the staircase method, with considerations given to fatigue life and fatigue limit at different reliability levels. Subsequently, the gears were characterized using optical microscopy and a microhardness tester to examine their metallographic structure and determine their hardness. The results indicate that the bending fatigue limits corresponding to gear reliability levels of 50%, 90%, and 99% are 390.00 MPa, 372.55 MPa, and 358.32 MPa, respectively. It was also observed that higher gear life stability corresponds to a lower sustainable fatigue limit stress. The analyses further reveal that under low loads, the main crack exhibits a relatively straight and smooth propagation trajectory, formed through the slow extension of an existing crack, whereas under high loads, the main crack displays a rough and serrated appearance, arising from the coalescence of microcracks initiated around graphite nodules.

## 1. Introduction

Driven by the strategic goals of “carbon peaking and carbon neutrality”, explicitly designates breakthroughs in core material technologies for lightweight and high-reliability transmission systems as a key objective. In new energy vehicles, high-efficiency transmission systems help reduce energy loss and enhance power transmission efficiency [1]. As a critical component of electric drive systems, the bending fatigue performance of gears directly influences transmission efficiency and limits the overall performance of the vehicle [2]. Therefore, studying the bending fatigue of gears is essential for improving the reliability of transmission systems and advancing technologies in sectors such as new energy vehicles. Aus China’s New Energy Vehicle Industry Development Plan (2021–2035) [3] tempered ductile iron (ADI) is an advanced ductile iron material produced through the heat treatment of conventional ductile cast iron. Driven by growing application demands, the production of ductile cast iron has been steadily increasing. With a stable supply of raw materials, the ADI industry continues to exhibit strong and sustained growth trends [4]. Compared with conventional ductile iron and standard forged steel, ADI offers superior noise reduction, reduced weight, excellent vibration damping, and lower production costs, making it particularly well-suited to the gear manufacturing requirements of new energy vehicles [5].

Bending fatigue has been extensively studied as a fundamental bottleneck limiting the safety and reliability of high-performance gears [6]. Hong et al. [7] developed a testing method to evaluate gear-bending fatigue life under two loading conditions and designed an adaptive diagnostic approach to terminate the tests. The fatigue tests conducted confirmed the effectiveness of the proposed method. Gabriel et al. [8] conducted a standard fatigue life assessment and carried out two sets of dual-stress-amplitude single-tooth bending fatigue tests on carburized gear steel to empirically investigate the impact of multistage loading on fatigue life. Multiple cumulative damage fatigue models were used to predict the fatigue life of specimens subjected to dual stress amplitudes, and the prediction accuracy of each model was assessed. Yan et al. [9] performed bending fatigue tests on carburized and shot-peened gears and analyzed fatigue crack propagation behavior. By examining the macromechanical properties and microstructural changes in the gear specimens, residual stress relaxation during bending fatigue was observed. Cular et al. [10] investigated the influence of gear module on the probability of subsurface bending fatigue failure using finite element analysis. Four gear modules were selected for the study, with fatigue performance and residual stress distribution kept constant. The results showed that a lower module reduces the likelihood of subsurface bending fatigue crack initiation in gears. Although considerable research has been conducted on gear-bending fatigue, experimental studies on ADI gears at the gear stage remain relatively limited.

The metallographic structure and hardness gradient of gears are critical factors in gear design and manufacturing, significantly affecting gear performance and service life [11]. To investigate the cause of early fractures in turbocharger gear teeth after prolonged operational cycles, Botvina et al. [12] conducted a systematic study of the chemical composition and microstructure of the gear material using metallographic analysis and microhardness testing. The analysis revealed that poor machining quality resulted in a hardened layer beneath the gear surface, within which structural defects were present. These defects initiated longitudinal microcracks, which served as origins for fatigue cracks. As the cracks propagated, they eventually led to the fracture of several gear teeth. Krishnasamy et al. [13] carried out a detailed metallurgical failure analysis on failed gears used in automotive engine cooling pumps. Metallographic examination showed that the internal structure of the gear consisted of a soft matrix composed of pearlite and ferrite. Cracks were found to originate at the fillet of the keyway and propagated toward the tooth root, clearly indicating that the failure was caused by fatigue fracture. Sun et al. [14] developed a novel three-dimensional numerical model incorporating hardness gradient and residual stress to predict the bending fatigue life of carburized gears. Single-tooth bending fatigue (STBF) tests were performed, and the results showed that the predicted fatigue life, failure location, and crack propagation path closely matched the experimental observations. Although extensive research has been conducted on gear performance through testing and characterization methods [15], the investigation of bending fatigue failure in ADI gears using these techniques still requires further in-depth study.

The present work focuses on investigating the bending fatigue performance of QTD 800 ADI gears and employs a combination of the conventional group method and the staircase variable load method to conduct bending fatigue tests. Fatigue life and limits at varying reliability levels are determined by fitting the data using several distribution functions, and the corresponding R–S–N curves for the gears are plotted accordingly. The gears are characterized using optical microscopy and a microhardness tester examine the microstructure and hardness of the investigated ADI gears. Additionally, the main crack characteristics of gears subjected to different load levels are assessed following failure. The present work provides support for the material selection of gears in new energy vehicle transmission systems and the industrial application of ADI gears.

## 2. Material and Methods

### 2.1. Material

The ADI employed in the present work has the following chemical composition, as presented in Table 1.

**Figure 1:**
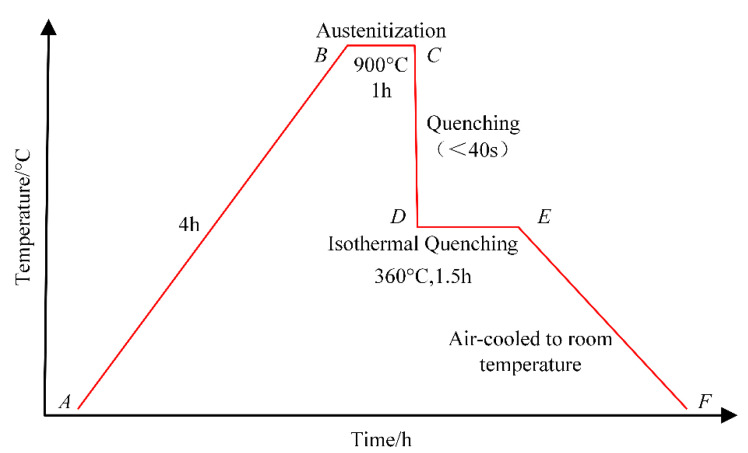
QTD 800 heat-treatment process.

The employed ADI is fabricated through an isothermal quenching heat-treatment process which consists of several steps, as indicated in Figure 1 [16], as follows:

Stage AB: the ADI is heated for 4 h to the austenitization temperature of 900 °C;
Stage BC: the ADI is heated at the austenitization temperature for 1 h to allow for a uniform transformation into carbon-enriched austenite;
Stage CD: the ADI is rapidly quenched in a salt bath maintained at the austempering temperature to quickly reach the desired temperature and prevent pearlitic transformation; this step is completed in less than 40 s;
Stage DE: the ADI is heated at the austempering temperature and maintained at 360 °C for 1.5 h;
Stage EF: the ADI is removed from the furnace and air-cooled at room temperature [17].

**Figure 2:**
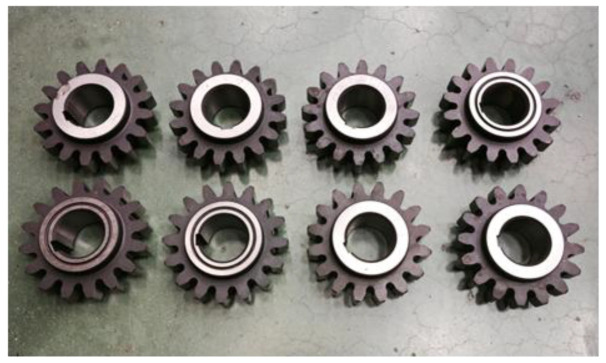
Test gears.

To investigate the performance of ADI materials, QTD-800-grade involute spur gears were manufactured using a designated process, as shown in Figure 2. Bending fatigue tests were then carried out to evaluate the bending fatigue performance of the ADI gears. The geometric parameters of the QTD 800 test gears are presented in Table 2.

### 2.2. Methods

#### 2.2.1. Characterization Techniques

In the study of gear-bending fatigue, characterization techniques are used to identify key features of the gear’s microstructure and microhardness, which significantly influence its bending fatigue performance [18]. Favorable surface properties can improve resistance to the initiation and propagation of fatigue cracks. By optimizing manufacturing processes, surface strengthening can be achieved, thereby extending gear service life and offering valuable guidance for enhancing the bending fatigue performance of ADI gears.

The metallographic microstructure of the samples was examined using the Axio Observer research-grade inverted universal metallographic microscope supplied by Beijing Precise Instrument Co., Ltd., China. This precision instrument, integrating optical microscopy, photoelectric conversion technology, and computer-image processing technology, achieves high-resolution and high-magnification imaging through optical principles. It magnifies the sample at high magnification using transmitted or reflected light, allowing for a clear display of the metallic material’s microstructure [19]. The specific steps for the metallographic examination of the gear samples are as follows:

(1) The cracked portion of the gear is sectioned along the critical cross-sectional line at the tooth root. A sample encompassing two tooth surfaces is selected to facilitate subsequent grinding, polishing, and microscopic examination;
(2) Coarse grinding of the sectioned tooth sample is performed using a bench grinder. The sample is held in a fixture and ground with uniform pressure in a single direction;
(3) Wet grinding is carried out using a metallographic pre-grinding machine, progressing from coarse to medium grit abrasive papers. The sample is held and ground with consistent pressure in one direction. After fine grinding, the sample is thoroughly cleaned;
(4) The sample is mechanically polished to a mirror finish using a metallographic polishing machine with a 0.05 μm diamond suspension. Appropriate pressure is applied throughout the process to ensure uniform contact between the sample and the polishing disc. Polishing fluid is continuously added to maintain moisture. After each polishing step, the sample is thoroughly cleaned to completely remove any residual polishing particles;
(5) The polished surface is etched with a 4% nitric acid alcohol solution for 10–15 s and then immediately rinsed with anhydrous ethanol and dried with air. This chemical treatment selectively dissolves or stains different phases or grain boundaries on the sample surface, enhancing contrast and clearly revealing the microstructure;
(6) The prepared sample is examined using an inverted universal microscope (Axio Observer). Initial low-magnification observation (50×–200×) is conducted to quickly scan the entire cross-section and assess the overall distribution of cracks and microstructure. Subsequently, medium-to-high magnification observation (200×–1000×) is performed to focus on the initiation, middle, and terminal regions of the root cracks, as well as the distribution and classification of graphite in the tooth surface and core.

**Figure 3:**
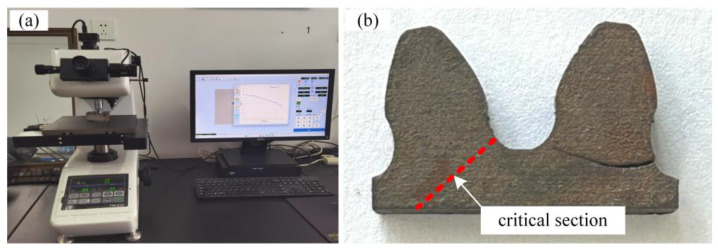
Characterization of gear hardness gradient: (**a**) microhardness testing device; (**b**) measurement positions.

The microhardness tester is a key instrument for characterizing the hardness gradient of gears, as shown in Figure 3. Its operating principle is based on the indentation hardness method, primarily employing the Vickers hardness measurement technique. A diamond square-pyramid indenter with an angle of 136° between opposite faces is used. Under a specified test load, the indenter is pressed into the surface of the specimen and held for a certain period. After the load is removed, an indentation is formed. The indentation is then observed under a microscope, and the lengths of its diagonals are measured. The microhardness value is calculated by referring to a standard table that correlates diagonal length with microhardness values. The calculation formula is as follows:

(1) $$HV=\frac{2F\sin68{}^{\circ}}{d^{2}}=1.8544\frac{F}{d^{2}}$$

In this context, HV denotes the Vickers hardness value, F is the applied test force, and d refers to the arithmetic mean of the indentation diagonal lengths, measured in millimeters (mm).

**Figure 4:**
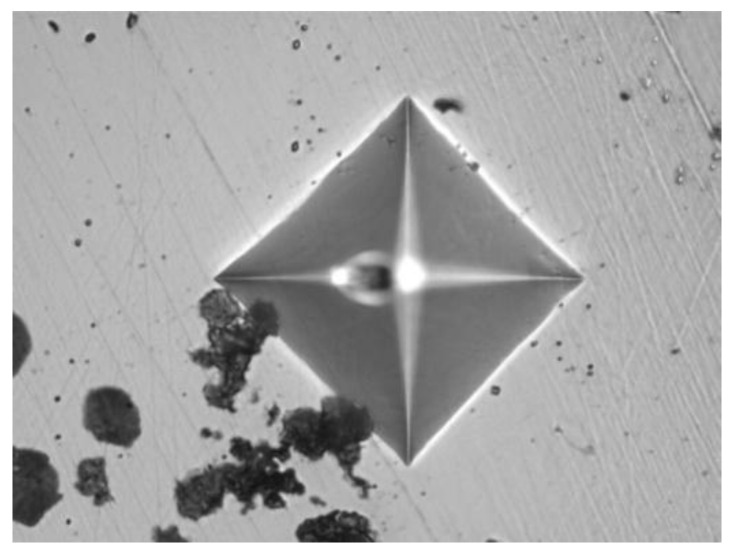
Indentation morphology.

The measurements were performed using the FUTURE-TECH FM-310 micro Vickers hardness tester from Japan, with a load of 25 gf and a dwell time of 15 s. The starting point was set at the critical cross-section of the tooth root, defined as the tangent point between the root profile transition curve and the 30° tangent line. Measurements were taken in the normal direction (perpendicular to the tooth surface), progressing from the surface toward the core. The measurement interval was 0.1 mm. A total depth of 1.5 mm was measured, encompassing the transition zone between the carburized layer and the substrate. The indentation morphology observed during the hardness test is shown in Figure 4., and the measured diagonal dimensions are listed in Table 3.

#### 2.2.2. Fatigue Testing Methods

**Figure 5:**
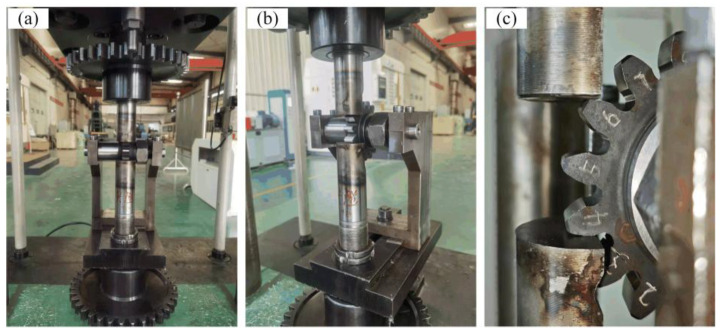
Bending fatigue testing apparatus: (**a**) test bench; (**b**) test fixture; (**c**) test gear.

The bending fatigue test of ADI gears serves as a crucial method for evaluating their fatigue resistance, as well as their stress conditions and fatigue life under actual operating conditions. To investigate the bending fatigue characteristics of ADI gears and obtain more accurate data, the GPS-200 high-frequency fatigue testing machine manufactured by Changchun Mechanical Science Research Institute Co., Ltd. was used for this test, as shown in Figure 5a. The performance parameters of the testing machine are listed in Table 4. To accommodate the tooth profile requirements of ADI gears, the test fixture was redesigned and improved. The loading hammer head was engineered with two concave surfaces. In the event of uneven load distribution along the tooth width during loading, the concave surfaces of the hammer head can deform to adjust the load distribution across the gear tooth width, thereby ensuring a uniform load distribution along the tooth direction, as illustrated in Figure 5b.

The QTD 800 gear fatigue test was carried out on a high-frequency fatigue testing machine using a single-tooth loading method to apply pulsating loads to the test gear. Pulsating loads were applied to the gear tooth at a frequency of 80–100 Hz, with a stress ratio of r=0.05 During the test, the alternating load was applied exclusively to the test tooth, and the test gear was not operated in meshing conditions. The test was terminated either when bending fatigue failure occurred on the gear tooth or when the number of cycles exceeded the specified threshold (3 × 10^6^). The number of stress cycles (fatigue life) under the applied stress was then recorded. According to the test standard GB/T 14230-2021 [20], a decrease in test frequency by approximately 5% (i.e., 5 Hz) is defined as the criterion for bending fatigue failure. The test was automatically terminated using the shutdown protection function of the testing machine. This bending fatigue test is classified as a high-cycle fatigue test. The testing procedure is conducted in accordance with the ASTM E466-15 [21] standard to ensure the scientific rigor of the experiment and the reliability of the results.

When determining the bending fatigue life of gears, the conventional group method is adopted because, compared with other testing methods, it allows for multiple tests to be conducted at the same stress level, resulting in relatively stable testing conditions, better repeatability, and higher reliability of the results [22]. Furthermore, it enables the collection of fatigue life data across different stress levels simultaneously, providing more comprehensive data. Therefore, the conventional group method is used to measure the fatigue life of ADI gears under various stress levels. In contrast, when determining the bending fatigue limit of gears, the staircase method is employed. By gradually adjusting the load, this method can approximate the fatigue limit with fewer test iterations. It also allows for real-time load adjustments based on the progress of the test, making it adaptable to different testing requirements and gear characteristics. Thus, the staircase method is selected to determine the bending fatigue limit of gears.

**Figure 6:**
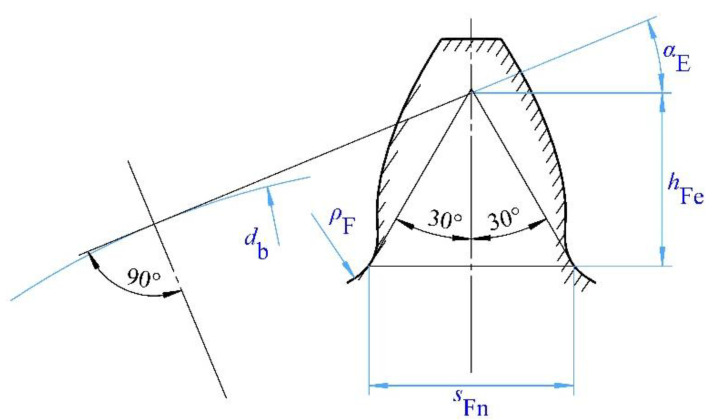
Load application diagram.

In this experiment, the dangerous section is determined using the 30° tangent method, with the load applied to the effective involute tooth surface near the tooth tip. The load application point is designated as E, as shown in Figure 6. The parameters obtained from Figure 6 are listed in Table 5.

According to GB/T 3480.3-2021 [23], “Calculation of Load Capacity of Spur and Helical Cylindrical Gears Part 3: Calculation of Root Bending Strength”, the root bending stress of the test gear is calculated by Equation (2), as follows:

(2) $${\sigma_{F}}^{\prime }=\frac{F_{t}\cdot Y_{FE}\cdot Y_{SE}\cdot Y_{\beta }\cdot Y_{B}}{b\cdot m_{n}\cdot Y_{ST}\cdot Y_{\delta relT}\cdot Y_{RrelT}\cdot Y_{X}}$$

$Y_{FE}$ is calculated as shown in Equation (3), as follows:

(3) $$Y_{FE}=\frac{\frac{6h_{Fe}}{m_{n}}\cdot \cos\left(\alpha_{E}\right)}{{\left(\frac{s_{Fn}}{m_{n}}\right)}^{2}\cdot \cos\left(\frac{\alpha \pi }{180}\right)}$$

The formula for calculating the stress correction factor, $Y_{SE}$ is as follows:

(4) $$Y_{SE}=\left(1.2+0.13L\right)q_{s}^{\frac{1}{1.21+\frac{2.3}{L}}}$$

where

(5) $$L=\frac{s_{Fn}}{h_{Fe}}$$

(6) $$q_{s}=\frac{s_{Fn}}{2\rho_{F}}$$

The helix angle β at the pitch circle is 0°. By referring to the diagrammatic table of the helix angle coefficient, it can be determined that $Y_{\beta }=1.0$. For external gears, the support ratio $\frac{S_{R}}{h_{t}}>1.2$. Therefore $Y_{B}=1.0$, $Y_{ST}=2.0$, $Y_{\delta relT}=0.998$, $Y_{RrelT}=0.998$, and $Y_{X}=1.0$ are selected according to ISO 6336-3:2019 [24].

In summary, the parameter values for Equation (2) are presented in Table 6.

Due to the limitations of the testing machine, the cyclic characteristic coefficient in the single-tooth loading test is 0.05. Therefore, the actual root stress must be converted into the pulsating cyclic root stress corresponding to a coefficient of 0, which is calculated using Equation (7).

(7) $$\sigma_{F}=\frac{(1-r)\cdot {\sigma_{F}}^{\prime }}{1-r\frac{{\sigma_{F}}^{\prime }}{\sigma_{b}+350}}$$

The calculated bending fatigue strength results are presented in Table 7.

**Figure 7:**
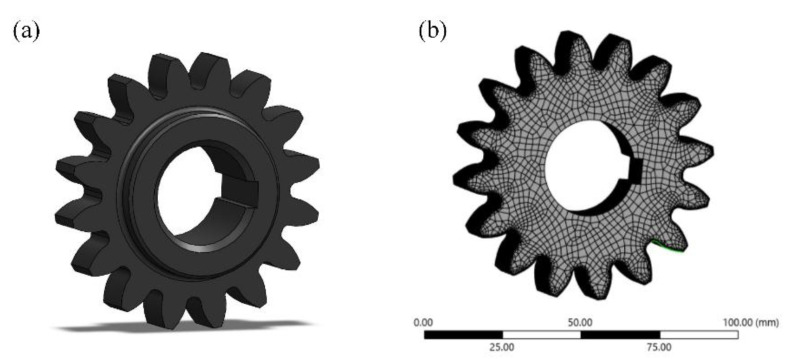
QTD 800 gear model diagram: (**a**) gear model; (**b**) gear mesh partition.

**Figure 8:**
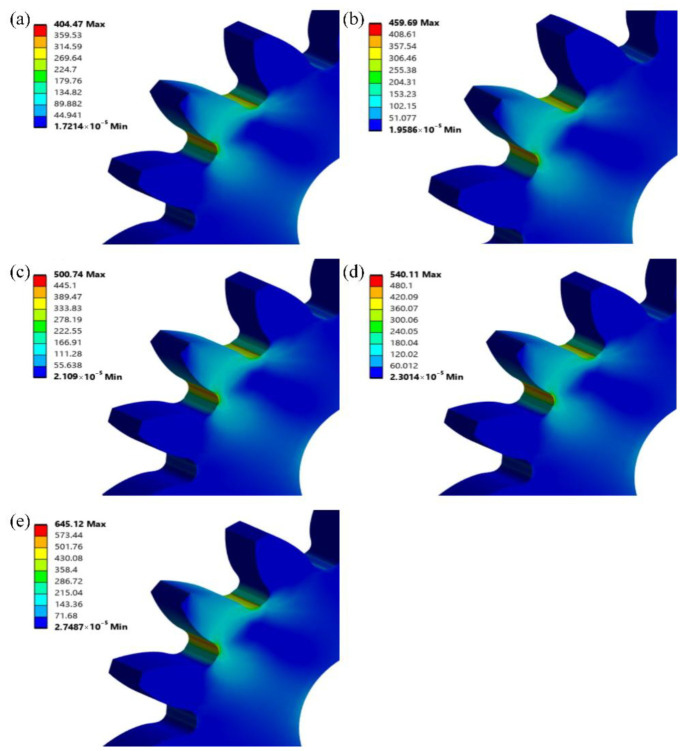
Stress contour maps at various load levels: (**a**) 14.4 kN; (**b**) 16.4 kN; (**c**) 17.7 kN; (**d**) 19.3 kN; (**e**) 22.9 kN.

Accurate calculation of root bending stress is essential for ensuring the reliability of gear-bending fatigue test results. To verify the accuracy of the theoretical calculation of root bending stress, the finite element analysis (FEA) method is applied. In this study, a three-dimensional model of the QTD 800 gear was created using SolidWorks 2020 software, as shown in Figure 7a, and the root bending stress is simulated using ANSYS Workbench 2022 R1. During the FEA process, material properties must be accurately defined based on actual conditions. The material parameters for QTD 800 are listed in Table 8. To enhance the computational accuracy of the model, the mesh size is set to 2 mm for the main body of the gear and 0.1 mm for the tooth surface during meshing, as shown in Figure 7b. Constraints and boundary conditions were applied to the inner bore surface of the gear based on actual experimental conditions. After the analysis conditions for the gear’s bending fatigue strength were defined, the model was solved. Numerical simulations were then performed to generate stress contour plots at the gear teeth under five different load levels, as shown in Figure 8.

The root bending stresses obtained from finite element simulation and theoretical calculations are presented in Table 9. As shown in the table, the maximum discrepancy between the root bending stresses from the finite element simulation and those calculated theoretically based on the experimental scheme is 0.2%, which meets engineering requirements. Therefore, the accuracy of the experimental scheme is confirmed.

## 3. Results and Discussion

### 3.1. Characterization Result Analysis

#### 3.1.1. Metallographic Analysis

**Figure 9:**
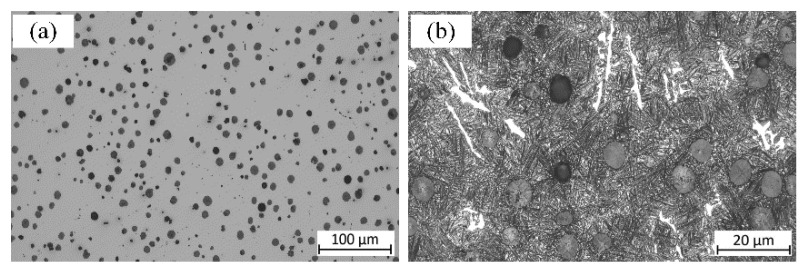
Microstructure of QTD 800: (**a**) before etching; (**b**) after etching.

Figure 9a, presenting the microstructure of QTD 800 before etching, shows the distribution of the graphite nodules. The nodules with a quasi-consistent shape are found to be uniformly distributed, and no significant agglomeration is observed.

Based on the image analysis, the characteristics of the graphite nodules (i.e., average diameter and roundness) are determined. The average roundness (R) of the graphite nodules was calculated using the following formula: $R=4\pi S/p^{2}$, where p is the perimeter and S is the area of the graphite nodules. It is found that the average diameter of the graphite nodules is 18.95 μm, and the average roundness of the graphite nodules is around 0.995. Most of the graphite appears small and round, indicating the effective control of graphite nodules, which can significantly reduce its influence on crack initiation.

Figure 9b presents the microstructure of QTD 800 after etching. It is characterized by a typical austenite–ferrite matrix. The ferrite, appearing in an acicular form, is densely and uniformly arranged, while the graphite nodules are embedded within the austenite phase.

#### 3.1.2. Microhardness Analysis

**Figure 10:**
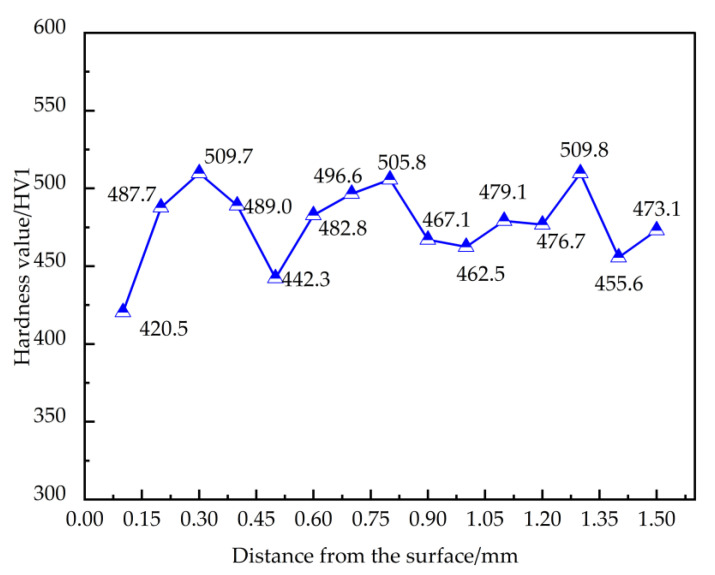
QTD 800 hardness gradient.

The hardness gradient distribution curve of the QTD 800 gear is shown in Figure 10. As seen in the figure, the measured surface hardness of the gear is 420.5 HV1. At various depths beneath the surface, the hardness shows significant fluctuations, indicating a non-uniform hardness gradient distribution. This considerable variation can mainly be attributed to the relatively small size of the indentation used in the hardness test, which makes the measurements highly sensitive to the substantial hardness differences between austenite and ferrite phas, resulting in fluctuating hardness values. Additionally, since the average spacing of graphite nodules is comparable to the indentation size, the presence and distribution of graphite also directly influence the local hardness measurements. Based on these findings, the hardness of the gear can be represented by the average hardness value, which is approximately 477.22 HV1. This suggests that the employed heat-treatment processes have no significant influence on the surface layer of QTD 800 gear.

### 3.2. Fatigue Test Results Analysis

A total of five stress levels are selected using the conventional group method for the QTD 800 gear-bending fatigue test, among which $\sigma_{F1}=402\;MPa$, $\sigma_{F2}=457\;MPa$, $\sigma_{F3}=495\;MPa$, $\sigma_{F4}=541\;MPa$, and $\sigma_{F5}=644\;MPa$. Each stress level includes at least five data points, with outliers occurring at the lower stress levels. The corresponding bending fatigue life cycles for the five stress levels are presented in Table 10.

The data obtained through the conventional grouping method are processed and analyzed. For each given stress level, n test points are conducted, and the corresponding fatigue life data are arranged in ascending order. The empirical distribution function of the fatigue life is expressed by Equation (8), as follows:

(8) $$P\left(N_{L}\right)=\frac{i-0.3}{n+0.4}$$

In the formula, n denotes the total number of test points, and i indicates the position of a test point in the sequence.

The fatigue test data were analyzed using distribution tests based on the normal distribution, lognormal distribution, and two-parameter Weibull distribution, and the corresponding fitted values of the distribution functions were calculated according to Equations (9)–(11).

(9) $$\Phi^{-1}\left[P(N_{L})\right]=\frac{1}{\sigma_{N}}(N_{L}-\mu_{N})$$

(10) $$\Phi^{-1}\left[P(N_{L})\right]=\frac{1}{\sigma_{\ln N}}(\ln N_{L}-\mu_{\ln N})$$

(11) $$\ln\ln\frac{1}{1-P(N_{L})}=\beta (\ln N_{L}-\ln\eta )$$

The linearized data were fitted using the least squares method, and the goodness of fit was assessed by the linear correlation coefficient, r The fitting followed the linear model Y=A+BX, and the fitted parameter values are presented in Table 11.

As shown in Table 11, when the bending fatigue test data of QTD 800 are fitted using different distribution functions, the two-parameter Weibull distribution exhibits a consistently stronger linear correlation than both the normal and lognormal distributions, indicating the best overall fit. Therefore, the two-parameter Weibull distribution is identified as the optimal life distribution function for the bending fatigue life of QTD 800 gears. Accordingly, the two-parameter Weibull distribution function is selected to determine the R–S–N curve. The shape and scale parameters are calculated based on the linear model parameters of the two-parameter Weibull distribution, and the results are shown in Table 12.

The reliable lifespans at different reliability levels are calculated using Equation (12), as shown in Table 13.

(12) $$N_{L.R}=\eta {\left(\ln\frac{1}{R}\right)}^{\frac{1}{\beta }}$$

The R–S–N curve is used to represent the mathematical relationship between tooth root bending stress (S) and fatigue life (N) at different reliability levels. The most commonly used form is the exponential function, as follows:

(13) $$S^{m}\cdot N=C$$

In the equation, m denotes the exponent, and C represents the constant in the S–N curve equation.

Taking the logarithm of both sides of the equation results in the following:

(14) mlgS+lgN=lgC

Based on Equation (14), the data in Table 13 were analyzed to determine the failure life of the gear under various stress conditions, as shown in Table 14.

Y=lgS, X=lgN, $B=-\frac{1}{m}$, and $A=\frac{lgC}{m}$. The R–S–N equation can be obtained by applying the least squares method to perform linear fitting on the data points at each stress level with the same reliability, as shown in Table 15.

**Figure 11:**
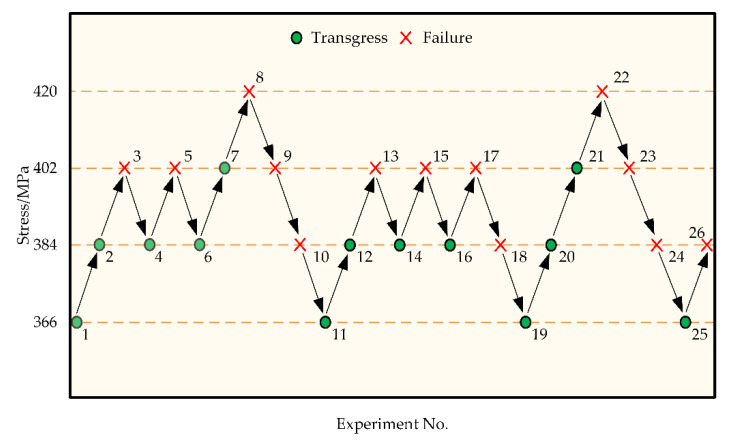
Lift–load variation test records.

Four load levels were selected for the ascending and descending variable load test, and the corresponding bending stresses were as follows: 420 MPa, 402 MPa, 384 MPa and 366 MPa. The experimental records are shown in Figure 11.

The test results obtained using the up-and-down variable load method are analyzed by treating “failure” as the event of interest. The numbers of failures and non-failures are then counted and calculated, as shown in Table 16.

The mean and standard deviation of stress are calculated using the formulas provided in Equations (15) and (16), respectively.

(15) $$\mu_{\sigma }=\sigma_{0}+∆\sigma \left(\frac{A}{N}-\frac{1}{2}\right)$$

(16) $$s_{\sigma }=1.62∆\sigma \left(\frac{NB-A^{2}}{N^{2}}+0.029\right)$$

The calculated average stress is $\mu_{\sigma }=390\;N/{mm}^{2}$, and the standard deviation is $S_{\sigma }=13.62\;N/{mm}^{2}$.

The fatigue limit stress at reliability R is calculated using Equation (17).

(17) $$\sigma_{R}=\mu_{\sigma }+\Phi^{-1}\left(1-R\right)s_{\sigma }$$

The calculated fatigue limit stresses for different reliabilities are shown in Table 17.

**Figure 12:**
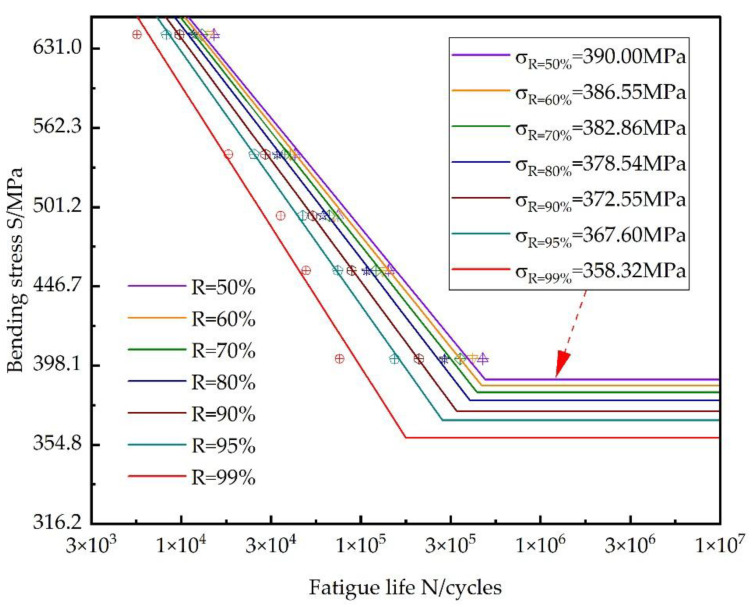
Fitted R–S–N curve of the QTD 800 gear.

Figure 12 shows the complete R–S–N curve for the QTD 800 gear.

Based on the fitted R–S–N equation, at a 50% reliability level, the fatigue life of the QTD 800 gear is estimated to be 477,077 cycles at low stress level I; 74,860 cycles at medium stress level III; and 15,199 cycles at high stress level V. At a 99% reliability level, the fatigue life of the QTD 800 gear is estimated to be 76,187 cycles at low stress level I; 35,813 cycles at medium stress level III; and 5676 cycles at high stress level V. The bending fatigue stress limit levels corresponding to bending fatigue reliability levels of 50%, 90%, 95%, and 99% are 390.00 MPa, 372.55 MPa, 367.60 MPa, and 358.32 MPa, respectively. It follows that the higher the bending fatigue limit capacity the gear can withstand, the lower its stability.

**Figure 13:**
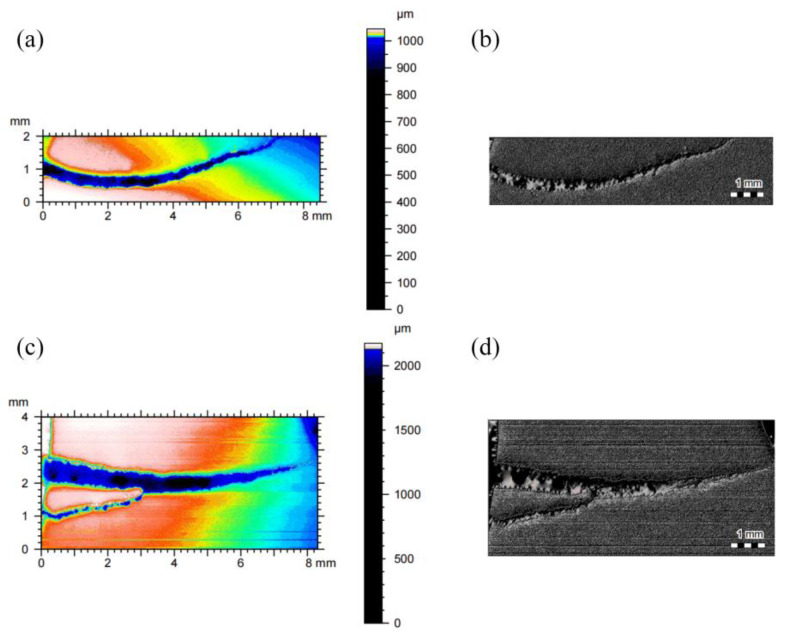
Three-dimensional crack morphology maps: specimen A—(**a**) surface three-dimensional height map and (**b**) surface crack morphology micrograph; specimen B—(**c**) surface three-dimensional height map and (**d**) surface crack morphology micrograph.

Crack analysis was carried out on gear specimens after bending fatigue tests under both low- and high-stress levels. A 3D profilometer was used to characterize specimen A, which exhibited cracks under a load of 14.4 kN, and specimen B, which exhibited cracks under a load of 22.9 kN, as shown in Figure 13. As shown in the figure, the main crack length under the low-stress level is approximately 7.4 mm, while under the high-stress level, it is about 8 mm. Secondary crack initiation was also observed, with a secondary crack length of approximately 3.1 mm. These results indicate that crack initiation is more likely to occur under high-stress levels.

**Figure 14:**
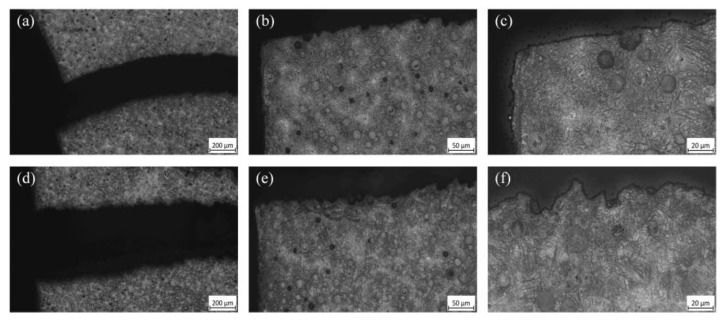
Metallographic images at the crack tips: specimen A—(**a**) 50×, (**b**) 200×, and (**c**) 500×; specimen B—(**d**) 50×, (**e**) 200×, and (**f**) 500×.

Metallographic examinations were performed on gear specimens subjected to low (c.f. Figure 14a–c) and high (c.f. Figure 14d–f) load levels. The microstructures were observed under magnifications of 50×, 200×, and 500×, as presented in Figure 14. It can be seen that the damage characteristics of these gears differ significantly under low- and high-load levels. Under low-stress cyclic loading, the main crack displays a relatively small opening width, with a consistently narrow and uniform profile, minimal variation along its path, and a relatively straight and smooth propagation trajectory; the main crack propagating slowly along the interface between the crack and the graphite nodules. As the crack advances under cyclic loading, no notable formation of microcracks is observed near the graphite nodules. When the main crack approaches these graphite nodules, they are gradually displaced, leaving behind smooth-edged voids which, however, do not serve as significant initiation sites for new cracks. In contrast, under high-stress cyclic loading, the main crack exhibits a substantially wider opening, with an overall profile marked by uneven width and pronounced fluctuations. The crack propagates rapidly along an irregular macroscopic path, with rough and jagged edges, due to the coalescence of microcracks initiated around graphite nodules; cyclic loading weakens the interfacial bonding between the austenite–ferrite matrix, which behaves as a composite material, and graphite nodules, favoring the early-stage microcrack initiation around graphite nodules, as reported by Ref. [25].

## 4. Conclusions

The bending fatigue performance of ADI gears fabricated using an austempering heat-treatment process is investigated. The microstructure and hardness of the gear specimens were characterized using optical microscopy and a microhardness tester. Bending fatigue tests were conducted, and the conventional group method was applied to determine the bending fatigue life at various reliability levels. Based on the results of the staircase variable load method, the fatigue limits corresponding to different reliability levels were calculated. The main crack characteristics of gears subjected to high- and low-load levels were also investigated following failure. A comprehensive bending fatigue performance curve of the gears was then established.

(1) The average hardness value of the QTD 800 gear measured using a microhardness tester was found to be 477.22 HV1. Additionally, metallographic analysis was performed on gear samples before and after etching. Before etching, the microstructure exhibited a uniform spheroidal graphite morphology. After etching, the microstructure revealed a typical austenite–ferrite matrix, with ferrite uniformly and densely distributed and spheroidal graphite embedded within the austenitic phase. Metallographic examinations performed on gears subjected to low- and high-loading conditions showed that under low-load fatigue testing, the main crack had a smaller opening width and propagated slowly along a relatively straight and smooth path. However, under high-load fatigue testing, the main crack exhibited a significantly wider opening, with an overall uneven width and rough, serrated edges; the main crack is formed by the coalescence of microcracks initiated around spheroidal graphites.
(2) The bending fatigue strength of ADI spur gears was investigated and calculated using the conventional grouping method and the up-and-down variable load method. A series of grouped bending fatigue tests were conducted, and the resulting experimental data were further processed using mathematical statistics and reliability theory. Based on the principles of reliability theory, the R–S–N curve for bending fatigue strength was fitted. The results showed that the bending fatigue stress limits corresponding to reliability levels of 50%, 90%, 95%, and 99% were 390.00 MPa, 372.55 MPa, 367.60 MPa, and 358.32 MPa, respectively. The experimental data revealed that as the stability of ADI gear life increases, the fatigue limit stress the gear can endure decreases. These findings provide a reliable foundation for the reliability-based design of the fatigue life of ADI gears.

## Figures and Tables

**Table 1:** Chemical composition of QTD 800.

Chemical composition	C	Si	Mn	P	S	Mo	Ni	Cu
Measured value/%	3.68	2.62	0.18	0.024	0.013	0.17	0.56	0.77

**Table 2:** Geometrical parameters of the specimen gears.

Parameter	Symbol	Numerical Value
normal module/mm	$m_{n}$	4.5
number of teeth	z	16
pressure angle/°	α	20
tooth width/mm	b	14
addendum modification coefficient	$h_{a}$	1
reference diameter/mm	$d_{w}$	72
tip diameter/mm	$d_{a}$	82.635
addendum modification factor	x	0.1817

**Table 3:** Indentation diagonal size.

Serial Number	Length of Diagonal 1/mm	Length of Diagonal 2/mm	Average Length/mm
1	66.44	66.36	66.40
2	61.39	61.91	61.65
3	59.97	60.64	60.31
4	60.92	62.23	61.58
5	64.86	64.61	64.74
6	62.34	61.60	61.97
7	61.23	60.96	61.10
8	60.44	60.64	60.54
9	63.28	62.71	63.00
10	63.13	63.50	63.32
11	61.55	62.87	62.21
12	62.18	62.55	62.37
13	60.29	60.33	60.31
14	64.07	63.50	63.79
15	62.65	62.55	62.60

**Table 4:** Performance specifications of the GPS-200 high-frequency fatigue testing machine.

Maximum Load/kN	Maximum Alternating Load/kN	Average Load Fluctuation Degree	Fluctuation of Alternating Load	Test Frequency Range/Hz
200	100	±0.5% F.S	±0.5% F.S	80–250

**Table 5:** Gear tooth parameter table.

Serial Number	Coefficient	Symbol	Numerical Value
1	Tooth thickness at the root danger cross-section/mm	$s_{Fn}$	8.2
2	Bending moment arm length/mm	$h_{Fe}$	7.23
3	Tooth root fillet radius/mm	$\rho_{F}$	2.6
4	Load application angle/°	$\alpha_{E}$	30.68
5	Base circle diameter/mm	$d_{b}$	67.658

**Table 6:** Tooth root bending stress calculation parameters.

Serial Number	Coefficient	Symbol	Numerical Value
1	E-Point Involute Coefficient	$Y_{FE}$	2.665
2	E-Point Stress Correction Factor	$Y_{SE}$	1.551
3	Coefficient of Spiral Angle for Bending Strength Calculation	$Y_{\beta }$	1.00
4	Flange Coefficient	$Y_{B}$	1.00
5	Stress Correction Factors Associated with the Dimensions of Standard Test Gears	$Y_{ST}$	2.00
6	Relative Tooth Root Fillet Sensitivity Factor	$Y_{\delta relT}$	0.998
7	Relative Surface Condition Factor of the Gear Tooth Root at Endurance Life	$Y_{RrelT}$	0.988
8	Bending Strength Size Factor	$Y_{X}$	1.00
9	Tensile Strength of Gear Material/MPa	$\sigma_{b}$	800

**Table 7:** Bending fatigue stress at the gear tooth root.

Payload $F_{t}$/kN	${\sigma_{F}}^{\prime }$/MPa	$\sigma_{F}$/MPa
14.4	415	402
16.0	461	447
16.4	471	457
17.0	490	476
17.7	509	495
19.0	548	533
19.3	555	541
22.0	634	619
22.9	659	644

**Table 8:** QTD 800 material parameters.

Parameter	Numerical Value
Density	7200 kg/m^3^
Elastic modulus	1.75 × 10^11^ Pa
Poisson’s ratio	0.28
Tensile strength	800 MPa
Yield strength	550 MPa

**Table 9:** Comparison table of root bending stress.

$F_{t}$/kN	Simulated Value/MPa	Theoretical Value/MPa	Error/%
14.4	405	402	−0.7
16.4	460	457	−0.6
17.7	501	495	−1.1
19.3	540	541	0.2
22.9	645	644	−0.1

**Table 10:** Bent-tooth bending fatigue life data.

Stress Level	Bending Stress/MPa	Number of Cycles	Vibration Frequency/Hz
I	402	236,060	96.2
		298,225	95.9
		465,346	96.6
		547,292	96.1
		580,425	96.7
		799,792	96.4
		3,000,000	95.3
II	457	100,783	95.7
		112,816	96.2
		153,878	96.4
		170,605	97.0
		171,021	96.1
III	495	54,892	95.9
		72,112	96.8
		75,854	97.1
		81,969	96.5
		85,716	96.2
IV	541	30,224	95.6
		41,868	96.3
		42,551	97.3
		47,289	98.2
		51,449	97.6
V	644	11,002	98.5
		12,299	97.8
		15,232	98.3
		17,688	97.0
		19,259	97.5

**Table 11:** Constants and linear correlation coefficients of the fitting equations.

Serial Number		I	II	III	IV	V
normal distribution	B	4.2344 × 10^−6^	2.4611 × 10^−5^	6.9004 × 10^−5^	1.0458 × 10^−4^	2.4488 × 10^−4^
	A	−2.0658	−3.4904	−5.1138	−4.4630	−3.6967
	r	0.9825	0.9357	0.9518	0.9605	0.9844
log-normal distribution	B	1.9106	3.2871	4.6257	4.0250	3.5999
	A	−24.8710	−38.9163	−51.8153	−42.8497	−34.5596
	r	0.9790	0.9323	0.9314	0.9385	0.9823
two-parameter Weibull distribution	B	2.3078	4.0067	5.7421	4.9562	4.2985
	A	−30.5420	−47.9262	−64.8126	−53.2543	−41.7567
	r	0.9835	0.9502	0.9668	0.9664	0.9808

**Table 12:** Characteristic parameters of the two-parameter Weibull distribution.

Serial Number	I	II	III	IV	V
shape parameter *β*	2.3078	4.0067	5.7421	4.9562	4.2985
scale parameter *η*	559,193	156,610	79,794	46,397	16,552

**Table 13:** Constant stress life at different reliability levels for the two-parameter Weibull distribution.

Reliability R	I	II	III	IV	V
0.50	477,077	142,920	74,860	43,090	15,199
0.60	417,977	132,437	70,985	40,516	14,157
0.70	357,730	121,081	66,680	37,684	13,022
0.80	291,941	107,706	61,450	34,281	11,676
0.90	210,899	89,310	53,922	29,464	9806
0.95	154,388	74,623	47,569	25,481	8294
0.99	76,187	49,682	35,813	18,340	5676

**Table 14:** Gear failure life at various stress levels.

Stress S/MPa	*lgS*	*R* = 0.5	*R* = 0.6	*R* = 0.7	*R* = 0.8	*R* = 0.9	*R* = 0.95	*R* = 0.99
402	2.6042	5.6786	5.6212	5.5536	5.4653	5.3241	5.1886	4.8819
457	2.6599	5.1551	5.1220	5.0831	5.0322	4.9509	4.8729	4.6962
495	2.6946	4.8742	4.8512	4.8240	4.7885	4.7318	4.6773	4.5540
541	2.7332	4.6344	4.6076	4.5762	4.5351	4.4693	4.4062	4.2634
644	2.8089	4.1818	4.1510	4.1147	4.0673	3.9915	3.9188	3.7541

**Table 15:** R–S–N fitting curve related parameters.

Reliability R/%	Exponent *m*	Constant *C*	Correlation Coefficient r
50	7.3314	5.0390 × 1024	0.9928
60	7.1839	1.8503 × 1024	0.9946
70	7.0225	6.1963 × 1023	0.9965
80	6.8166	1.5230 × 1023	0.9984
90	6.5147	1.9233 × 1022	0.9999
95	6.2539	3.1600 × 1021	0.9990
99	5.7937	1.1830 × 1020	0.9854

**Table 16:** Process parameters used to calculate the mean and standard deviation of stress.

Stress/MPa	Stress Level i	f_i_	i f_i_	i^2^ f_i_
420	2	2	4	8
402	1	7	7	7
384	0	4	0	0
process parameters		N=13	A=11	B=15
		$\frac{NB-A^{2}}{N^{2}}=0.438>0.3$		

**Table 17:** Bending fatigue limit stress at different reliability levels.

**Reliability *R***	0.50	0.60	0.70	0.80	0.90	0.95	0.99
**Fatigue Limit Stress** $\sigma_{R}/MPa$	390.00	386.55	382.86	378.54	372.55	367.60	358.32

## Data Availability

The original contributions presented in this study are included in the article. Further inquiries can be directed to the corresponding author.

## Nomenclature

**Symbol**	**Meaning**	**Unit**
b	Tooth width	mm
d	Arithmetic mean of the diagonal lengths of the indentations	mm
$d_{a}$	Addendum circle diameter	mm
$d_{b}$	Base circle diameter	mm
$d_{w}$	Pitch circle diameter	mm
$F_{t}$	Apply load	N
HV	Vickers hardness value	—
$h_{a}$	Addendum coefficient	—
$h_{Fe}$	Bending moment arm length	mm
$m_{n}$	Normal module	mm
$N_{L}$	Number of tooth root stress cycles	—
p	Graphite sphere circumference	μm
R	Graphite roundness	—
	Reliability	—
r	Correlation coefficient	—
S	Graphite spherule surface area	μm^2^
	Stress	N/mm^2^
$s_{Fn}$	Tooth thickness at the root danger cross-section	mm
$s_{\sigma }$	Standard deviation of stress in the load–unload Method	N/mm^2^
x	Dislocation coefficient	—
$Y_{B}$	Flange coefficient	—
$Y_{FE}$	E-point profile coefficient	—
$Y_{RrelT}$	Relative surface condition factor of gear tooth root at Endurance Life	—
$Y_{SE}$	E-point stress correction factor	—
$Y_{ST}$	Stress correction factors associated with the dimensions of standard test gears	—
$Y_{X}$	Flexural strength size factor	—
$Y_{\beta }$	Helical angle coefficient for calculating bending strength	—
$Y_{\delta relT}$	Relative Tooth Root Fillet Sensitivity Factor	—
z	Number of teeth	—
α	Pitch circle pressure angle	(°)
$\alpha_{E}$	Load Application Angle	(°)
β	Helical angle	(°)
	Shape Parameter of the Weibull Distribution	—
η	The scale parameter of the Weibull distribution function	—
$\rho_{F}$	Root fillet radius	mm
$\mu_{\sigma }$	Mean value in step-load method	N/mm^2^
$\sigma_{b}$	Tensile strength of gear material	MPa
$\sigma_{F}$	Gear root stress	MPa
${\sigma_{F}}^{\prime }$	Root stress is observed when the cyclic characteristic coefficient r ≠ 0.	MPa
$\sigma_{R}$	Bending fatigue limit stress of the tooth root at reliability R	MPa
